# The Effects of Temperature on the Growth, Survival, and Feeding of *Chrysaora pacifica* (Cnidaria: Scyphozoa) Ephyrae

**DOI:** 10.3390/biology15080597

**Published:** 2026-04-09

**Authors:** Kyong-Ho Shin, Keun-Hyung Choi

**Affiliations:** 1 Department of Earth, Environmental and Space Sciences, Chungnam National University, 99 Daehak-ro, Yusung-gu, Daejeon 34134, Republic of Korea; shin1987@hanwha.com; 2 Aqua Planet Corporation, Ltd., 300 Gwanggyohosugongwon-ro, Yeongtong-gu, Suwon-si 16514, Republic of Korea

**Keywords:** *Chrysaora pacifica*, ephyra stage, scyphozoan jellyfish, temperature effects, feeding dynamics, survival rate, environmental change, East Asian waters

## Abstract

*Chrysaora pacifica*, a scyphozoan jellyfish widely distributed in East Asian waters, has recently shown signs of range expansion along the coasts of Korea, Japan, and China. However, ecological information on its early planktonic stage, the ephyra, remains limited. In this study, we experimentally investigated the effects of seawater temperature on the growth, feeding, and survival of *C. pacifica* ephyrae under controlled laboratory conditions. Five temperature treatments (12, 16, 20, 24, and 28 °C) were selected based on the species’ natural occurrence period. The results showed that ephyrae exhibited stable growth and feeding at 20–24 °C, with a high survival rate of approximately 90%, indicating that this range represents the optimal thermal condition for the ephyra stage. At 28 °C, growth and feeding were highest among all treatments; however, survival declined sharply to 22.5%, suggesting that elevated temperature may impose physiological stress. In contrast, at 12 °C, both growth and feeding activity were markedly reduced, and survival decreased to 32.5%. These findings demonstrate that temperature is a key environmental factor influencing the physiological performance and survival of *C. pacifica* ephyrae. This study provides essential baseline data for understanding the early life-stage ecology of this species and contributes to improving predictions of jellyfish population dynamics and potential distribution shifts in East Asian marine ecosystems under future environmental change.

## Simple Summary

Jellyfish blooms are becoming increasingly frequent in many coastal marine ecosystems worldwide and can have substantial impacts on fisheries, tourism, and marine ecosystem dynamics. In East Asian waters, the scyphozoan jellyfish *Chrysaora pacifica* has recently been reported to be expanding its distribution range; however, ecological information on its early life stages remains limited. In particular, little is known about how environmental factors affect the planktonic ephyra stage, which plays a crucial role in the development of adult jellyfish populations. In this study, we investigated the effects of seawater temperature on the growth, feeding, and survival of *Chrysaora pacifica* ephyrae under controlled laboratory conditions. Our results showed that ephyrae exhibited the most stable growth, feeding activity, and survival at 20–24 °C, suggesting that this temperature range may represent the optimal thermal environment for the ephyra stage of this species. Although higher temperatures promoted growth and feeding, survival decreased markedly at 28 °C. These findings provide new ecological insights into the early life stage of *Chrysaora pacifica* and offer baseline data to support future monitoring, prediction, and management of jellyfish blooms under changing environmental conditions. In particular, this study contributes to improving our ability to anticipate jellyfish population dynamics and potential distribution shifts in East Asian marine ecosystems in response to climate change.

## 1. Introduction

Globally, increases in atmospheric carbon dioxide caused by human activities and the resulting climate change are exerting diverse effects on marine environments and ecosystems [1,2,3,4,5]. Representative examples include rising sea surface temperatures and observed increases in biodiversity within regional marine ecosystems [5,6,7,8]. Among these factors, rising sea temperatures have been suggested as a key environmental driver of jellyfish blooms [9,10,11,12,13]. Several studies have reported that elevated temperatures can markedly increase asexual reproduction during the polyp stage of the jellyfish life cycle, particularly by enhancing budding activity and influencing strobilation timing under specific thermal conditions [3,14,15]. In addition, experimental studies have shown that higher temperatures can promote ephyral growth, with growth rates increasing under warmer conditions [11,16,17]. However, the relationship between rising sea temperatures and jellyfish bloom dynamics remains poorly understood and requires further research and empirical evidence [18,19].

Jellyfish blooms can have multifaceted impacts on human economic activities [19,20]. These impacts include the deterioration of harvested seafood quality [4], negative effects on the tourism industry [21], and operational disruptions at power plants caused by jellyfish clogging intake systems [22]. Conversely, jellyfish are also commercially utilized as food products in some regions [23] and play important ecological roles in marine food webs, including contributions to water quality regulation [24]. Moreover, jellyfish are known to serve as key prey for marine megafauna, including sea turtles, some of which are classified as internationally endangered species [25].

**Figure 1:**
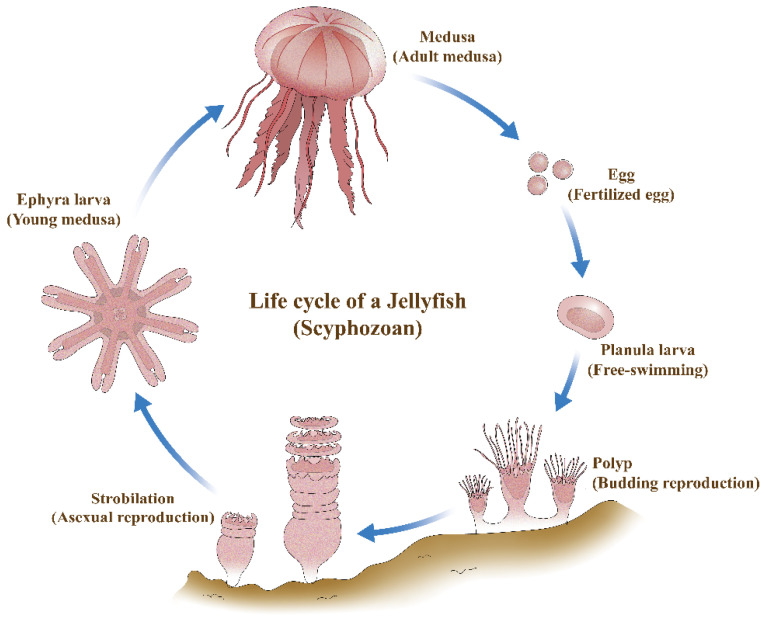
Life cycle of the scyphozoan jellyfish *C. pacifica*, which alternates between a sexually reproducing medusa stage and an asexually reproducing polyp stage. Fertilized planula larvae settle and develop into benthic polyps, which proliferate and release ephyrae through strobilation. The ephyra stage, which is influenced by environmental factors such as temperature, subsequently develops into adult medusae, thereby completing the life cycle. Arrows indicate the progression of the life cycle.

Jellyfish species responsible for large-scale blooms vary among regional marine ecosystems; however, most bloom–forming species belong to the class Scyphozoa [26,27]. The life cycle of scyphozoans predominantly involves sexual reproduction through the fusion of male and female gametes [28]. Females release fertilized eggs into the water column, where they develop into planula larvae (Figure 1). After a brief planktonic phase, the planula settles on a suitable substrate and transforms into a benthic polyp. Polyps can increase in number through various forms of asexual reproduction, including binary fission and budding. In response to environmental cues and physical stimuli, polyps undergo strobilation, a process of transverse fission, during which they release free-swimming ephyrae into the water column. These ephyrae subsequently grow and differentiate into adult medusae.

Among the scyphozoan jellyfish known to cause large–scale blooms in East Asian waters, particularly around Korea, Japan, and China, *Aurelia coerulea* and *Nemopilema nomurai* are the most widely reported species [27,29,30,31,32]. However, in recent years, *Chrysaora pacifica* (Cnidaria: Scyphozoa), a member of the genus *Chrysaora* [33], has also been reported to be expanding its distribution range across East Asian marine ecosystems [31,34,35,36,37].

Species of the genus *Chrysaora* are widely distributed across marine environments worldwide and exhibit considerable taxonomic diversity [34]. Species within this genus are distinguished by a range of morphological and developmental characteristics, including body coloration, tentacle number, radial canal morphology, sequence of tentacle development, and other morphometric traits, which vary according to geographic distribution and life history strategy [38].

*C. pacifica*, a species distributed in East Asian waters, belongs to the phylum Cnidaria, class Scyphozoa, and order Semaeostomeae. Prior to 2010, it had been classified as *Chrysaora melanaster* [39]. However, following morphological and taxonomic revisions within the genus *Chrysaora*, it was reclassified as a distinct species, *C. pacifica*, separate from *C. melanaster* [38]. In Korea, the first record of *C. pacifica* was reported by Park [40]. In Japan, the species was initially documented in the North Pacific during the 1970s [41], and natural polyps were first discovered on the seafloor of Sagami Bay in 2011 [39]. More recently, the first occurrences of *C. pacifica* in China were reported from Bohai Bay in 2018 and 2019 [37].

Compared with other scyphozoan species, such as *A. coerulea* and *N. nomurai*, *C. pacifica* appears to exhibit population outbreaks less frequently in East Asian waters [35,42]. However, this species is considered hazardous to human activities because of its potent venom [34,43]. Recent studies have reported range expansion of *C. pacifica* populations in East Asia, driven by the movement of genetically and geographically distinct lineages [35,37]. These findings suggest that *C. pacifica* may undergo genetic differentiation among populations under the influence of environmental factors [35], and that its continued range expansion could pose a potential threat to the structure and functioning of marine ecosystems in the East Asian region [44]. Several studies have examined the effects of environmental factors on the ecology of *C. pacifica* during the benthic polyp stage [39,45,46]. However, research on how environmental conditions affect this species during its early planktonic ephyra stage remains extremely limited. Understanding the ecological responses of *C. pacifica* ephyrae to environmental variables is essential, as such knowledge provides a fundamental basis for predicting future jellyfish bloom events involving medusa populations [47,48,49].

Accordingly, based on the natural occurrence period of *C. pacifica* in East Asian waters [34,37], this study evaluated the effects of seawater temperature, a major environmental factor, on the (1) growth, (2) feeding, and (3) survival of *C. pacifica* ephyrae. We expected that, as temperature increased, *C. pacifica* ephyrae would exhibit stable growth and survival, whereas the lowest growth and survival would occur under low-temperature conditions. However, considering the experimental findings of Toyokawa [39], which indicated that low temperatures can induce strobilation in *C. pacifica* polyps, we hypothesized that the ephyrae of this species would retain a certain capacity for growth and survival even under low-temperature conditions.

In this study, *C. pacifica* ephyrae were cultured under a range of temperature conditions to quantitatively assess the effects of temperature on growth, feeding, and survival, and to determine the thermal range within which this species can most stably persist. The resulting data enable us to evaluate how seawater temperature may influence the potential spread of *C. pacifica* populations in East Asian waters and how such expansion could interact with blooms of other scyphozoan jellyfish. By focusing on the early planktonic stage, this study provides essential ecological insights into *C. pacifica* and offers baseline data that may serve as useful ecological indicators.

## 2. Materials and Methods

### 2.1. Collection and Cultivation of C. pacifica Polyps

To obtain *C. pacifica* ephyrae for the present experiment, polyps were obtained from the Port of Nagoya Public Aquarium (Nagoya, Aichi Prefecture, Japan). These polyps originated from fertilized eggs produced through artificial insemination of male and female medusae collected from the Port of Nagoya (35°05′27.5″ N, 136°52′37.1″ E). The polyps were subsequently transported to the jellyfish research facility at Aqua Planet Gwanggyo Aquarium in Suwon, Republic of Korea, where they were maintained in incubators (MyTEMPTM Mini, H2200-HC, Benchmark Scientific, Sayreville, NJ, USA) at a constant temperature of 22 ± 0.5 °C. The rearing temperature for *C. pacifica* polyps was determined based on the optimal culture conditions reported by Toyokawa [39]. Artificial seawater was prepared using Red Sea Salt (Public Aquarium Part A; Red Sea, Ltd., Eilat, Israel) and adjusted to a salinity of 33 PSU. Prior to use, the seawater was subjected to a three-stage filtration process consisting of a sand filter (first stage), a bag filter (second stage), and a housing filter (third stage). Polyp culture followed the protocol described by Gambill and Peck [50], except that no aeration was provided. Except during feeding and water exchange, the polyps were maintained under constant dark conditions in the incubator. In this study, feeding was conducted once every three days using *Artemia franciscana* (SEP-ART, INVE Aquaculture, Dendermonde, Belgium) that had been nutritionally enriched with Spirulina for 2 h. To obtain ephyrae for the experiment, the polyps were maintained under continuous culture for approximately three months, during which they underwent asexual reproduction via podocyst formation.

### 2.2. Induction of Strobilation and Collection of C. pacifica Ephyrae

Strobilation in *C. pacifica* polyps was induced to obtain ephyrae using the temperature-reduction method described by Toyokawa [39]. Polyps maintained in incubators at a stable temperature of 22 ± 0.5 °C were subjected to a stepwise cooling protocol, in which the temperature was reduced by 2 °C per day over five consecutive days until it reached 12 ± 0.5 °C. Strobilation was first observed approximately 11 days after the onset of temperature reduction (Video S1). Ephyrae released from the polyps were collected daily for five consecutive days until a sufficient number for the experiment was obtained. The collected ephyrae were maintained and cultured in Breeding Air Kreisel tanks measuring 310 × 330 × 110 mm (W × H × D), manufactured by Schuran Seawater Equipment BV (Aalsmeer, The Netherlands). The ephyrae were evenly distributed among ten separate Breeding Air Kreisel tanks, each equipped with a gentle aeration system to maintain a stable swimming environment. To prevent mortality caused by abrupt environmental changes, the pre–experimental rearing conditions were maintained at the same temperature and salinity as those of the environment from which the ephyrae had been released (temperature, 12 ± 0.5 °C; salinity, 33 PSU). A cooling system (Model DBA-075, Dae-Lim Co., Busan, Republic of Korea) was used to regulate water temperature. No food was provided to the ephyrae during this pre-experimental holding period.

### 2.3. Experimental Assessment of Growth, Survival, and Feeding in C. pacifica Ephyrae

**Figure 2:**
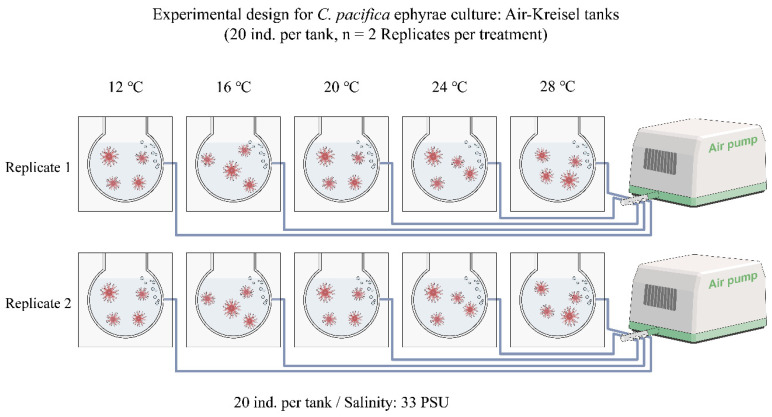
Experimental setup for culturing *C. pacifica* ephyrae. Ephyrae were reared in Air–Kreisel tanks under five temperature conditions (12, 16, 20, 24, and 28 °C) at a constant salinity of 33 PSU. Each temperature treatment consisted of two replicate tanks (*n* = 2), with 20 individuals per tank.

For this experiment, Breeding Air Kreisel tanks were used as experimental units and were set up in duplicate under five temperature conditions (12, 16, 20, 24, and 28 °C), all maintained at a constant salinity of 33 PSU (Figure 2). The environmental conditions were designed to reflect the natural temperature and salinity profiles observed during the seasonal occurrence period (May to September) of *C. pacifica* in East Asian waters.

Temperature data for the coastal waters of Korea and Japan were obtained from the official website of the National Institute of Fisheries Science (https://www.nifs.go.kr), which provides raw sea surface temperature data from the National Oceanic and Atmospheric Administration (NOAA). Data from two consecutive years (2023–2024), covering the months from May to September, were analyzed, revealing typical temperature ranges of 16–24 °C in May and June, 24–28 °C in July and August, and approximately 28 °C in September. In addition, sea temperature data for Chinese coastal waters were based on temperature records reported during the occurrence of *C. pacifica* in Bohai Bay by Wang et al. [37], indicating a range of 17–26 °C in June and July. The lowest experimental temperature condition, 12 °C, was established based on the strobilation-inducing temperature reported by Toyokawa [39]. Salinity data were incorporated from raw datasets provided by the Ocean Climate Prediction Center (OCPC, https://www.ocpc.kr), reflecting conditions in East Asian waters over two consecutive years (2023–2024) during the months from May to September. The average salinity during this period was 33 PSU.

Each experimental unit contained 20 *C. pacifica* ephyrae, and each temperature treatment consisted of two replicate tanks, resulting in a total of 40 individuals per treatment. Because the availability of newly released *C. pacifica* ephyrae at a comparable developmental stage was limited, the experiment was conducted under controlled laboratory conditions with two replicate tanks per temperature treatment. To ensure stable acclimatization to the target temperatures, the ephyrae were initially maintained under baseline conditions (temperature, 12 ± 0.5 °C; salinity, 33 PSU). Subsequently, water temperature was gradually adjusted over five days using a cooling system at a rate of 2–3.2 °C per day until the predefined target temperature for each treatment was reached.

The experimental period lasted 20 days, a duration determined based on the study by Shin and Choi [44], which evaluated growth, survival, and feeding in ephyrae of the scyphozoan species *Sanderia malayensis*. The seawater used in this experiment was prepared using the same artificial seawater product, Red Sea Salt (Public Aquarium Part A; Red Sea, Ltd.), as described in Section 2.1. Feeding was conducted once daily using *A. franciscana* enriched with Spirulina for 2 h. The feeding regime followed an excess food provision approach, as recommended by Shin and Choi [51] and Riisgård [52] for laboratory-based ephyra culture studies. This approach was used to prevent food limitation and inter-individual competition among the ephyrae, thereby minimizing potential external factors that could affect the experimental outcomes.

Water quality in each experimental unit was maintained by replacing 100% of the water every two days with freshly prepared artificial seawater adjusted to the designated temperature and salinity conditions of each treatment.

During the experimental period, ephyrae growth, feeding, and survival were measured at 2-day intervals over a total of 10 time points. Growth, feeding, and survival measurements followed the procedures described by Shin and Choi [44]. At each sampling point, ten ephyrae were randomly selected from each treatment and observed under an optical microscope (Olympus SZX2–ILLK, Tokyo, Japan). Individual-level measurements of growth (mm) and feeding activity (number of prey items observed in the gastric cavity per individual after 1 h) were recorded.

**Figure 3:**
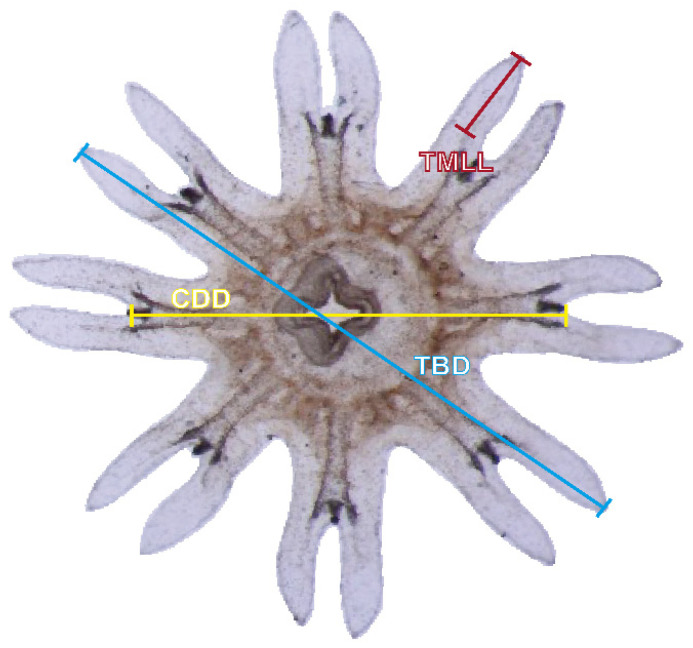
Measurement points and reference criteria used to assess growth in *C. pacifica* ephyrae, adapted from Straehler-Pohl and Jarms [53]. Red, yellow, and blue lines indicate the measurement ranges for each section. Ephyra size was expressed as total body diameter (TBD, mm), calculated as the sum of the central disc diameter (CDD, mm) and total marginal lappet length (TMLL, mm). The image was captured using an Olympus SZX2-ILLK microscope (Olympus, Tokyo, Japan).

Ephyra size (mm) was measured following the method of Straehler-Pohl and Jarms [53], using total diameter, defined as the sum of the central disc diameter and marginal lappet length (Figure 3). To illustrate growth changes across treatments, the largest individual among the ten randomly sampled ephyrae per treatment was selected on days 1, 10, and 20 based on total diameter, and images were captured using an optical microscope. The specific growth rate of ephyrae (% d^−1^) was calculated using the following formula:

% *Growth day*^−1^ = *ln* [(*D2*/*D1*)^3^]/(*t2* − *t1*) × 100

This formula was originally proposed by Båmstedt et al. [47] and has been widely applied in previous studies on ephyra growth, including those of Widmer [48] and Shin and Choi [51].

In this equation, *D1* and *D2* represent the mean diameter of ephyrae at two consecutive sampling points. Measurements were conducted ten times throughout the experimental period, and the mean diameter at each time point was used for the calculation. The variables *t1* and *t2* indicate the time interval (days) between measurements.

Feeding activity was assessed by providing *A. franciscana* and recording the number of prey items observed in the gastric cavity of each individual after 1 h under an optical microscope. This measurement represents a proxy for short-term prey ingestion rather than a true ingestion or clearance rate. It was used as an individual-level proxy for feeding activity in subsequent statistical analyses.

Survival was assessed every two days during water exchange. Individuals exhibiting normal swimming behavior were recorded as surviving, whereas those showing abnormal or no swimming activity were considered non-viable and recorded as dead. Survival analyses were conducted using pooled individual data for each treatment. Although this approach is commonly used, it does not explicitly account for potential clustering effects within tanks, and this limitation should be considered when interpreting the results.

### 2.4. Statistical Analysis

This study employed generalized linear mixed models (GLMMs), as recommended by Bolker et al. [54], to analyze changes in the growth and feeding activity of *C. pacifica* ephyrae under five temperature conditions (12, 16, 20, 24, and 28 °C). The GLMMs analyses were based on individual-level growth and feeding activity data collected at 2-day intervals over a 20-day experimental period. At each sampling point, ten ephyrae were randomly selected and measured for each treatment.

The experimental unit in this study was the tank, with two replicate tanks established for each temperature treatment. At each sampling point, individual ephyrae were randomly selected from each tank, resulting in a hierarchical data structure in which individual observations were nested within tanks. All analyses were conducted using individual-level measurements rather than aggregated means. To account for the non-independence of individuals within the same tank, tank identity was included as a random effect in the models. In addition, repeated measures were not obtained from the same individuals over time; instead, different individuals were randomly sampled at each time point, and temporal variation was accounted for within the mixed-effects framework.

In the GLMMs framework, the dependent variables were growth (mm) and feeding activity (number of prey items observed in the gastric cavity per individual after 1 h), while the independent variables included temperature, experimental day, and their interaction (Temperature × Day). To account for temporal variation within experimental units, tank-specific random intercepts and random slopes for day were included in the models.

When significant main or interaction effects were detected, Tukey’s post hoc tests were performed to identify differences among treatments. All GLMM analyses were conducted using the lme4 package in R [55], and statistical significance was set at *p* < 0.05. Descriptive statistics are presented as mean ± standard deviation (SD). No additional data transformation was required.

In addition, linear regression analysis was performed to illustrate growth trajectories over time under each temperature treatment. Mean size (mm) values at each sampling point were used to fit regression lines for each temperature treatment, and the resulting regression equations and coefficients of determination (R^2^) were presented. This analysis was conducted for visualization purposes and was based on mean values, whereas the main statistical analyses were performed using individual-level data within the GLMMs framework.

Survival analysis of *C. pacifica* ephyrae was performed using the Kaplan–Meier method [56]. Survival was assessed at 2-day intervals over the 20-day experimental period based on the number of surviving and deceased individuals in each treatment, and the estimates were presented with 95% confidence intervals (CI). Differences among survival curves were evaluated using the log-rank test. Survival analyses were conducted using the survival [57] and survminer [58] packages in R. Analyses were based on pooled individual data for each treatment. Although this approach is commonly applied, it does not explicitly account for potential clustering effects within tanks, and this limitation should be considered when interpreting the results.

Differences in degrees of freedom among response variables reflect differences in the number of individual-level observations used in each analysis, rather than differences in data aggregation. In particular, the relatively large degrees of freedom observed in the growth analysis resulted from the inclusion of individual-level data across multiple time points within the mixed-effects framework.

## 3. Results

### 3.1. Effect of Temperature on the Growth of C. pacifica Ephyrae

**Figure 4:**
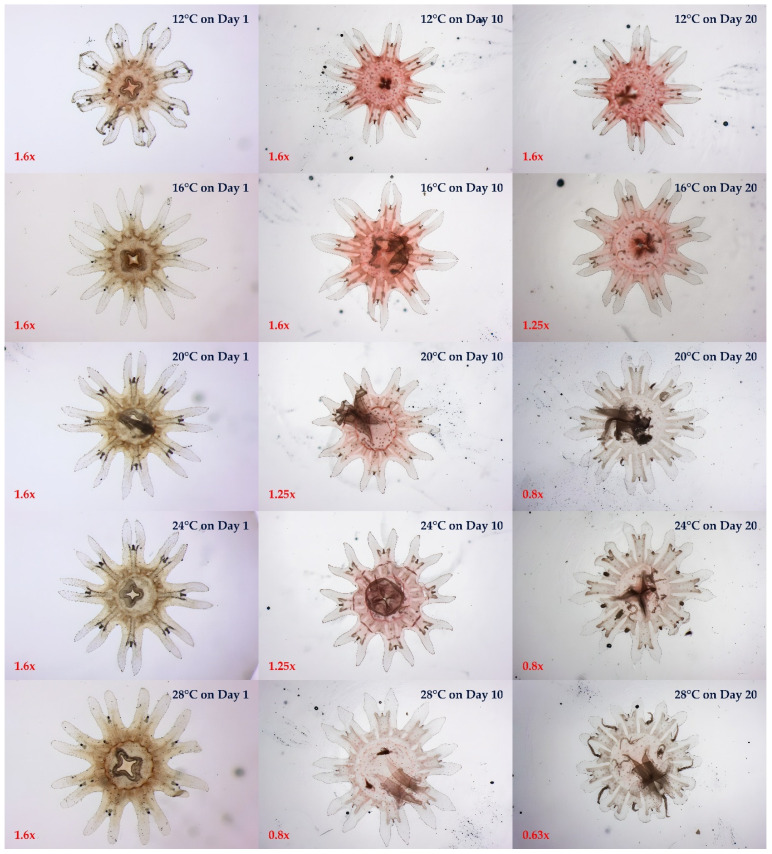
Morphological changes in *C. pacifica* ephyrae reared under five temperature conditions (12, 16, 20, 24, and 28 °C) at three time points (Days 1, 10, and 20). Representative images illustrate developmental progression and temperature-dependent differences in body size and morphology. Images were captured using an Olympus SZX2-ILLK microscope (Olympus, Tokyo, Japan).

Photographic records of growth changes in *C. pacifica* ephyrae cultured under five temperature conditions (12, 16, 20, 24, and 28 °C) on Days 1, 10, and 20 are shown in Figure 4. Growth and morphological development generally increased with temperature, with the greatest body size and lappet elongation observed at 28 °C on Day 20 (Figure 4). In contrast, ephyrae reared at 12 °C exhibited minimal growth and contracted lappets throughout the experimental period. At intermediate temperatures (20 and 24 °C), ephyrae showed relatively stable growth, although body size and morphological development were less pronounced than those observed at 28 °C (Figure 4).

The mean growth (mean ± SD) in each treatment was 2.60 ± 0.17 mm at 12 °C, 3.05 ± 0.39 mm at 16 °C, 3.44 ± 0.59 mm at 20 °C, 3.42 ± 0.61 mm at 24 °C, and 3.95 ± 0.81 mm at 28 °C (Table 1). The highest mean growth was observed at 28 °C (Table 1).

**Figure 5:**
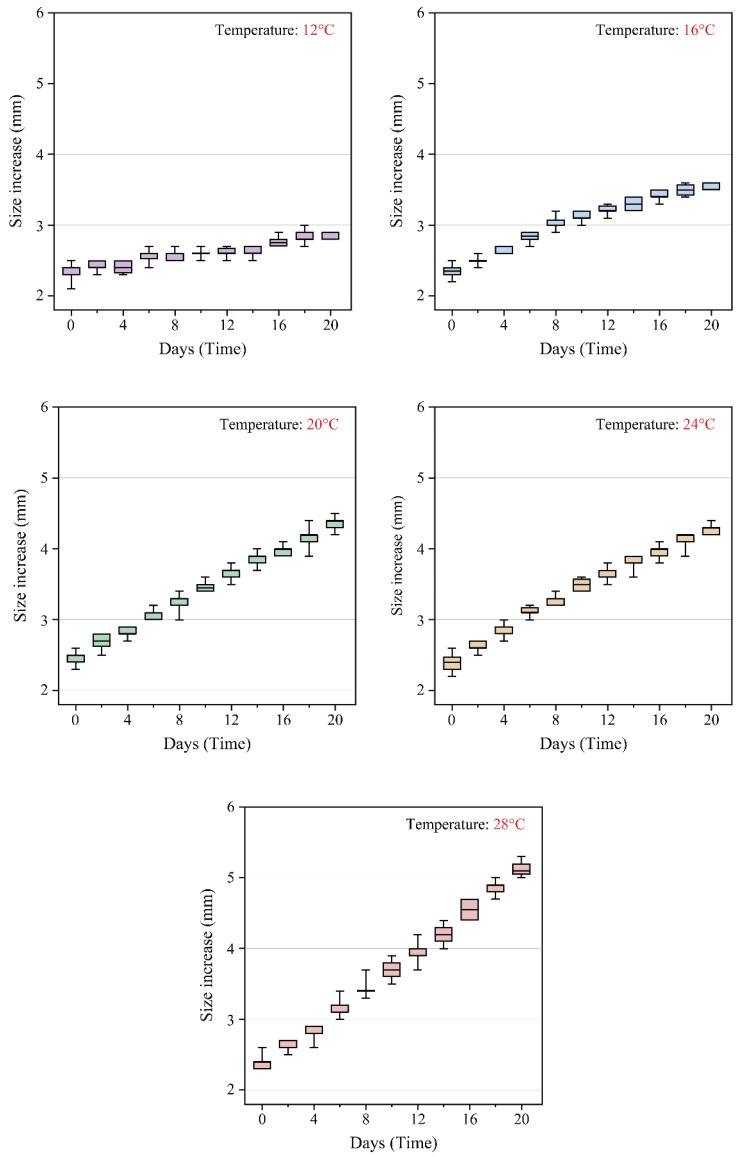
Temporal changes in size increase (mm) of *C. pacifica* ephyrae reared under five temperature conditions (12, 16, 20, 24, and 28 °C) over a 20-day period. Box plots show that ephyrae cultured at 28 °C exhibited the highest median and the widest interquartile range (IQR) among the temperature treatments. Individuals were randomly sampled from each replicate tank at each time point.

Growth changes (size increase) in *C. pacifica* ephyrae cultured under five temperature conditions are shown in the box plot in Figure 5. Across all temperature treatments, the box plots shifted upward over time, indicating progressive increases in body size throughout the experimental period, with the most pronounced increase observed at 28 °C. The 28 °C treatment exhibited the highest median and the widest interquartile range (IQR) among the temperature treatments (Figure 5). Descriptive statistics (mean ± SD) for each treatment are presented in Table 1.

**Figure 6:**
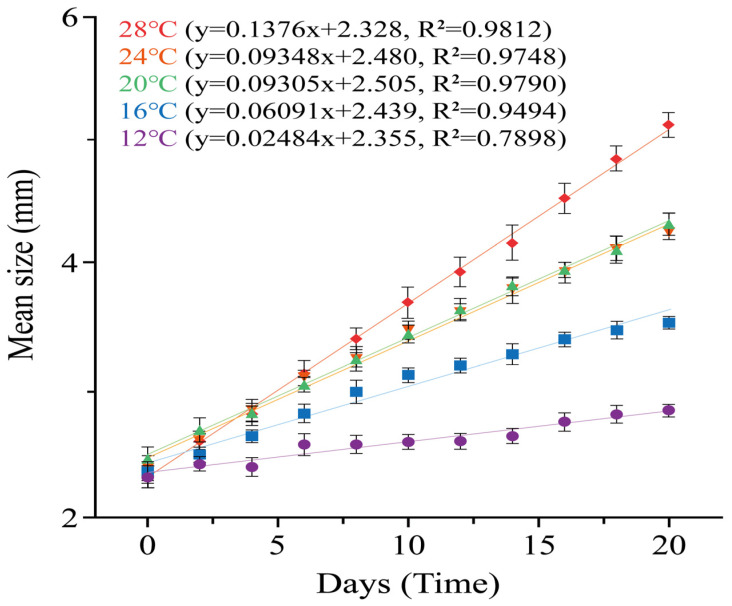
Linear regression plots illustrating temporal changes in the mean size (mm) of *C. pacifica* ephyrae under five temperature conditions (12, 16, 20, 24, and 28 °C) over a 20-day experimental period. Regression equations and coefficients of determination (R^2^) are shown for each temperature treatment. Error bars indicate standard deviations (SD).

Linear regression analysis based on mean size (mm) at each sampling point further illustrated growth trajectories over time under each temperature treatment (Figure 6). The steepest growth trajectory was observed at 28 °C (y = 0.1376x + 2.328, R^2^ = 0.9812), whereas the shallowest was observed at 12 °C (y = 0.02484x + 2.355, R^2^ = 0.7898). The regression slopes at 20 °C (y = 0.09305x + 2.505, R^2^ = 0.9790) and 24 °C (y = 0.09348x + 2.480, R^2^ = 0.9748) were similar, indicating comparable growth trajectories under these two temperature conditions. The 16 °C treatment showed an intermediate growth trajectory relative to the other treatments (y = 0.06091x + 2.439, R^2^ = 0.9494; Figure 6).

The mean specific growth rates (% d^−1^) of *C. pacifica* ephyrae cultured under five temperature conditions were 3.24% d^−1^ at 12 °C, 6.42% d^−1^ at 16 °C, 8.84% d^−1^ at 20 °C, 9.22% d^−1^ at 24 °C, and 12.1% d^−1^ at 28 °C (Table 2). The highest and lowest specific growth rates were observed at 28 °C and 12 °C, respectively (Table 2). Variability in body size measurements is presented as mean ± standard deviation (SD) in Table 1, whereas specific growth rate estimates are presented with 95% confidence intervals (CI) in Table 2.

The results of the generalized linear mixed model (GLMM) analysis of growth changes in *C. pacifica* ephyrae cultured under five temperature conditions showed that the main effect of time (Day) was statistically significant (β = − 0.0456, t = − 23.1814, *p* < 0.001; Table 3), indicating that growth changed significantly over time. The main effect of temperature was not statistically significant (β = 0.0012, t = 0.0773, *p* = 0.939; Table 3), suggesting that temperature alone did not have a significant overall effect on growth. However, the interaction between temperature and time (Temperature × Day) was statistically significant (β = 0.0064, t = 65.5922, *p* < 0.001; Table 3), indicating that the slopes of growth change over time differed significantly among temperature treatments.

The results of Tukey’s post hoc tests for the temperature × day interaction showed that the difference between 20 °C and 24 °C was not statistically significant from Day 8 onward (Table S1, *p* > 0.05). In contrast, all other temperature combinations showed statistically significant differences in the interaction effects through Day 20 (Table S1, *p* < 0.001). In addition, the 28 °C treatment showed significantly greater growth changes than the other temperature treatments (Table S1, *p* < 0.001).

### 3.2. Effect of Temperature on the Feeding Activity of C. pacifica Ephyrae

**Figure 7:**
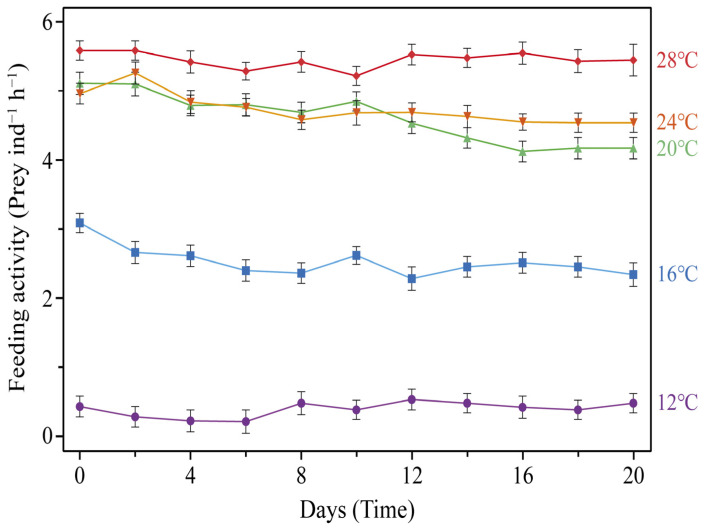
Mean feeding activity (prey ind^−1^ h^−1^) of *C. pacifica* ephyrae reared under five temperature conditions (12, 16, 20, 24, and 28 °C) over a 20-day period. Ephyrae reared at 28 °C showed the highest feeding activity, whereas those at 12 °C showed the lowest feeding activity. Error bars represent standard deviations (SD). Individuals were randomly sampled from each replicate tank at each time point.

The feeding activity of *C. pacifica* ephyrae cultured under five temperature conditions (12, 16, 20, 24, and 28 °C), expressed as the number of prey items observed in the gastric cavity per individual after 1 h of exposure, is presented in Figure 7. The mean values (mean ± SD) for each treatment were 0.41 ± 0.521 at 12 °C, 2.47 ± 0.644 at 16 °C, 4.47 ± 0.521 at 20 °C, 4.59 ± 0.738 at 24 °C, and 5.27 ± 0.521 at 28 °C (Table 4). The highest mean feeding activity was observed at 28 °C (Table 4). This metric represents a short-term gut-content proxy rather than a true ingestion or clearance rate.

The results of the generalized linear mixed models (GLMMs) analysis showed that the main effect of time (Day) on feeding activity was not statistically significant (β = 0.0001, t = 0.0070, *p* = 0.934; Table 5), indicating that this proxy did not vary significantly over time. In contrast, the main effect of temperature was statistically significant (β = 0.3041, t = 22.7419, *p* < 0.001; Table 5), indicating that feeding activity increased with increasing temperature. The interaction between temperature and time (Temperature × Day) was not statistically significant (β = −0.0010, t = −1.8738, *p* = 0.061; Table 5), although it approached significance at the α = 0.05 level.

To evaluate the main effect of temperature, Tukey’s post hoc comparisons showed no statistically significant difference between the 20 °C and 24 °C treatments (Table S2, *p* = 0.801). However, significant differences were observed among all other temperature treatments (Table S2, *p* < 0.001). The GLMM results indicated that feeding activity was significantly affected by temperature, and Tukey’s post hoc test further showed that the value at 28 °C was significantly higher than those observed under the other temperature conditions (Table S2, *p* < 0.001).

### 3.3. Effects of Temperature on the Survival Rate of C. pacifica Ephyrae

**Figure 8:**
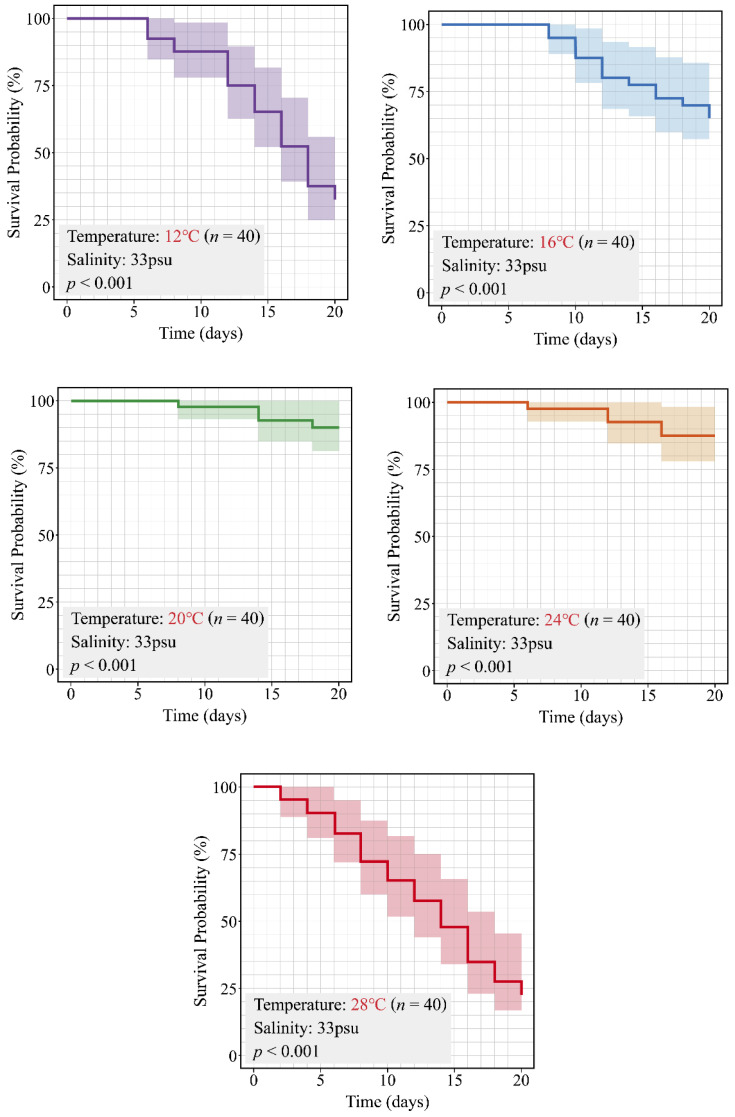
Kaplan–Meier survival curves of *C. pacifica* ephyrae reared under five temperature conditions (12, 16, 20, 24, and 28 °C) over a 20-day period. Survival probability was highest at 20 °C and 24 °C, whereas both low (12 °C) and high (28 °C) temperature conditions were associated with reduced survival. Shaded areas indicate 95% confidence intervals. Each temperature treatment consisted of two replicate tanks, with 20 individuals per tank. Survival analysis was based on pooled individual data for each treatment and did not explicitly account for potential clustering effects within tanks.

The Kaplan–Meier survival analysis of *C. pacifica* ephyrae reared under five temperature conditions (12, 16, 20, 24, and 28 °C) is shown in Figure 8. The observed survival rates were 32.5% at 12 °C (95% CI: 20.8–50.8%) and 65.0% at 16 °C (95% CI: 51.8–81.6%; Figure 8, Table 6). In contrast, relatively high survival rates were observed at 20 °C and 24 °C, reaching 90.0% (95% CI: 81.2–99.8%) and 87.5% (95% CI: 77.8–98.4%), respectively (Figure 8, Table 6). However, the 28 °C treatment showed the lowest survival rate among all treatments, with only 22.5% survival (95% CI: 12.7–40.0%; Figure 8, Table 6). The survival curves further indicated that survival probability remained relatively stable at 20 °C and 24 °C, whereas more pronounced declines were observed at 12 °C and 28 °C, with the 16 °C treatment showing an intermediate pattern (Figure 8).

To evaluate differences among survival curves across the five temperature treatments over the 20-day experimental period, a log-rank test was performed. The results showed statistically significant differences among the temperature treatments (χ^2^ = 70.614, df = 4, *p* < 0.001; Figure 8, Table 6), indicating that survival patterns differed significantly among temperature conditions.

## 4. Discussion

*C. pacifica* is a representative scyphozoan jellyfish occurring in East Asian waters and is primarily distributed along the coastal regions of Korea and Japan [31,34]. This species possesses potent nematocyst venom [34,43], and numerous cases of envenomation associated with human activities have been continuously reported [38,43,59,60]. Recent studies on *C. pacifica* have mainly focused on its taxonomic classification, population monitoring, and molecular ecological approaches related to its occurrence in regional marine ecosystems [31,34,35,36]. In addition, several laboratory studies have reported the effects of water temperature on asexual reproduction and transverse fission during the polyp stage of this species [39,45,46].

However, previous studies on the early planktonic ephyra stage of various scyphozoan species have shown that temperature significantly influences growth, feeding activity, and survival [14,17,44,48], whereas ecological studies focusing on the early planktonic ephyra stage of *C. pacifica* remain very limited.

The results of the present study showed that *C. pacifica* ephyrae exhibited the most stable growth, feeding activity, and survival at 20 °C and 24 °C. According to Tukey’s HSD post hoc test, no statistically significant difference was observed between these two temperatures. Notably, both treatments showed high survival rates approaching 90%, suggesting that the optimal temperature range for the stable growth and survival of *C. pacifica* ephyrae is approximately 20–24 °C. In contrast, the 28 °C treatment resulted in significantly higher growth and feeding activity than the other temperature treatments (*p* < 0.001). However, survival at 28 °C was the lowest, at 22.5%. These findings are consistent with those of Shin and Choi [44], who conducted experiments on *S. malayensis* ephyrae collected from Japanese waters. They reported that elevated temperature (28 °C) stimulated physiological activity and promoted growth and feeding, but excessive thermal stress significantly reduced survival during the ephyra stage. Therefore, the low survival rate of *C. pacifica* ephyrae observed at 28 °C in the present study is likely attributable to physiological stress induced by elevated temperature, which may have reduced survival relative to the other temperature treatments.

In the present experiment, the low-temperature condition of 12 °C resulted in the lowest growth and survival of *C. pacifica* ephyrae. Toyokawa [39] reported that strobilation in *C. pacifica* polyps was induced when temperature decreased from 22–23 °C to approximately 10 °C. In addition, field observations in Sagami Bay, Japan, recorded the occurrence of ephyrae of this species during the colder winter months (December–January), when seawater temperatures were relatively low [61,62]. Based on these previous findings, we expected that *C. pacifica* ephyrae would retain at least some capacity for growth and survival even under the lowest experimental temperature condition of 12 °C. However, feeding activity at 12 °C was the lowest among all temperature treatments, and both growth and survival were significantly lower than those observed at 16 °C (*p* < 0.001).

Meanwhile, Fu et al. [63] reported, in starvation resistance experiments using *Aurelia aurita* ephyrae, that individuals exposed to low-temperature conditions (9–15 °C) were able to survive without feeding for at least 33.8 days and up to 58.6 days. These findings suggest that *A. aurita* ephyrae released into environments with limited food availability, such as during winter, possess sufficient carbon reserves and metabolic capacity to withstand prolonged periods of food deprivation. In addition, Shin and Choi [44] reported that *S. malayensis* ephyrae exhibited significantly reduced feeding activity under the low-temperature condition of 15 °C, but maintained a relatively stable survival rate of 77%, suggesting that physiological tolerance may have contributed to survival under low-temperature conditions. In contrast, Fu et al. [17] suggested that low temperature suppresses the growth of *A. coerulea* ephyrae, whereas increasing temperature accelerates the growth of ephyrae and may subsequently promote bloom formation. Taken together, these previous studies suggest that the low growth and survival of *C. pacifica* ephyrae at 12 °C may reflect suppressed physiological and metabolic activity under low-temperature conditions, similar to the physiological stress observed at the high-temperature condition of 28 °C. In addition, *A. aurita*, as reported by Fu et al. [63], survived for 38.4 days under a low-temperature condition of 12 °C, whereas *C. pacifica* ephyrae in the present study showed a relatively low survival rate of 32.5% during the 20-day experimental period at the same temperature. However, direct comparisons of physiological tolerance to low temperatures between the two species may be limited because differences in life–history strategies, seasonal occurrence patterns, and ecological tolerance ranges may exist. Moreover, sufficient data for direct comparison are currently lacking. Therefore, the physiological tolerance of *C. pacifica* ephyrae observed at 12 °C in the present study may reflect physiological and ecological differences from species of the genus *Aurelia*.

At 16 °C, the growth, feeding activity, and survival of *C. pacifica* ephyrae were significantly lower than those observed at 20–24 °C (*p* < 0.001), but significantly higher than those recorded at 12 °C (*p* < 0.001). Although the present experiment was conducted under controlled laboratory conditions, these results may partially reflect early life-history patterns occurring in natural environments, given previous reports that polyps of this species initiate ephyra release during low-temperature periods from winter to early spring [39,45]. In particular, under very low-temperature conditions such as 12 °C, reduced metabolic activity may negatively affect feeding activity and survival in ephyrae. In contrast, ephyrae that persist under such low-temperature conditions may gradually recover metabolic activity as temperature increases to moderate levels, such as 16 °C, resulting in increased feeding activity and improved survival.

These findings are consistent with those reported by Fu et al. [17] for *A. coerulea* ephyrae and suggest that temperature variation is a key environmental factor influencing the growth and survival of *C. pacifica* during the ephyra stage. However, the results of the present study alone are insufficient to fully elucidate the physiological and ecological characteristics of this stage in the species. In addition, it should be noted that the present experimental design employed only two replicate tanks per treatment, with different individuals sampled repeatedly over time. This constraint primarily reflects the practical difficulty of securing a sufficient number of ephyrae at a comparable developmental stage under controlled laboratory conditions, and consequently indicates a limitation in the effective level of independent replication. Although the statistical analyses were conducted at the individual level, this limitation should be carefully considered when interpreting the results. As a consequence, the statistical models may overestimate the degree of independent information, and this should be taken into account when drawing broader ecological inferences. Therefore, future research should aim to improve methodological approaches for securing sufficient numbers of ephyrae under controlled laboratory conditions, while also incorporating a wider range of environmental factors, such as photoperiod, salinity variation, and prey availability, together with long-term field-based monitoring.

The effects of water temperature, as an important environmental factor, on the growth and survival of the early planktonic ephyra stage of scyphozoan jellyfish have been reported in various laboratory studies involving genera such as *Chrysaora*, *Aurelia*, and *Cyanea* [14,17,48,64,65]. Schäfer et al. [14] suggested that the optimal temperature range for stable growth and survival of *Aurelia solida* is approximately 15–20 °C. Fu et al. [17] reported that, in *A. coerulea*, the feeding and swimming rates of ephyrae increased with rising temperature. In contrast, Widmer [65] reported that *Aurelia labiata* exhibits a relatively broad temperature tolerance range of 12–21 °C for stable growth and swimming behavior, but noted that feeding activity decreases as temperature increases. Widmer [48] also reported that, in Northern Hemisphere *Cyanea* species, including *Cyanea capillata* and *Cyanea lamarckii*, the temperature range supporting stable growth was 4–9 °C. That study further suggested that rising seawater temperatures associated with climate change may facilitate shifts in the distribution of cold–water *Cyanea* species. In addition, laboratory studies on *Chrysaora* species have shown that *Chrysaora quinquecirrha*, which occurs in Chesapeake Bay, prefers temperatures above 20 °C for stable growth [64]. Widmer [48] also reported that *Chrysaora fuscescens* and *Chrysaora hysoscella* exhibited maximum growth rates at approximately 17 °C and 19 °C, respectively, suggesting that ocean warming may contribute to range expansion in these species.

The early life history of *C. pacifica* is characterized by polyp asexual reproduction via podocyst formation during the warmer summer months [45], followed by ephyra release during the cooler winter period [39,61,62]. In addition, several previous studies have reported that *A. coerulea* and *N. nomurai*, which also occur in East Asian waters, undergo strobilation under a broad range of environmental conditions, from the low-temperature winter period to the prey-rich spring and summer seasons [13,66,67,68], resulting in a relatively prolonged period of ephyra release [30]. Moreover, *A. coerulea* and *N. nomurai* exhibit high medusa population densities annually from May to September in East Asian waters [4,32], whereas *C. pacifica* shows significantly lower medusa population densities during the same period [31,35,42]. In other words, *C. pacifica* ephyrae, which are released primarily during winter when seawater temperature is relatively low and prey availability is limited, may experience greater constraints on survival during subsequent medusa population formation than *A. coerulea* and *N. nomurai*, which release ephyrae even during periods of higher temperature and greater prey availability.

These ecological characteristics during the early planktonic stage may also have important implications for population dynamics and trophic interactions during the medusa stage. Species of the genus *Chrysaora* are known to exhibit medusivorous behavior by preying on gelatinous plankton [48], and *C. pacifica* has been reported to prey on *A. coerulea* occurring during the same period using its potent nematocysts [69]. Meanwhile, Shin and Choi [44] reported that the optimal temperature range for stable growth and survival of *S. malayensis* ephyrae is approximately 20–24 °C. Avian et al. [70] further reported that *S. malayensis* ephyrae feed on gelatinous plankton using their potent nematocysts. Notably, the most stable temperature range for growth and survival of *C. pacifica* ephyrae identified in the present study was also 20–24 °C, which is consistent with the temperature range reported by Shin and Choi [44] for stable physiological responses in *S. malayensis* ephyrae. In addition, the natural occurrence period of *S. malayensis* ephyrae in Japanese waters has been reported to extend from May to August [71,72,73], which overlaps with the occurrence period of *C. pacifica* populations reported in East Asian waters, including South Korea, Japan, and China [31,35,37]. These findings suggest that the occurrence periods of these species may partially overlap in East Asian waters, and their simultaneous appearance in regional marine ecosystems could lead to interspecific competition for food resources. Moreover, *C. pacifica* is known to prey on *A. coerulea* populations occurring during the same period [69]. Potential food competition with *N. nomurai*, which seasonally migrates from Chinese coastal waters to Korean and Japanese waters, may also represent an important ecological factor in regional marine ecosystems [32]. However, quantitative field data on food competition and predatory interactions among scyphozoan jellyfish occurring in East Asian waters remain limited, and further studies incorporating gut content analysis and stable isotope analysis are needed.

The salinity conditions in this experiment were established based on the two-year average salinity data (2023–2024) for East Asian waters provided by the Ocean Climate Prediction Center (OCPC) of South Korea, and a constant salinity of 33 PSU was applied across all experimental treatments (Figure 2). Globally, increased precipitation associated with climate change has been identified as a major driver of frequent fluctuations in marine salinity [74,75]. Such salinity variability may influence physiological responses during the early planktonic ephyra stage of scyphozoan jellyfish [17,47,51,76] and is also closely related to adult population density and population dynamics [48,51]. Therefore, future research should evaluate the physiological and ecological responses of *C. pacifica* ephyrae under marine conditions in which salinity fluctuates frequently due to climate-related factors such as river discharge and precipitation, and should further identify the optimal salinity range for this species.

Climate change can facilitate the range expansion of scyphozoan jellyfish in regional marine ecosystems [77,78], potentially causing economic losses in fisheries and tourism, as well as alterations in biodiversity patterns [79]. Indeed, range expansions of *A. coerulea* and *N. nomurai* in East Asian waters in response to marine environmental change have been frequently reported [32,80,81], and occurrences of *C. pacifica* have also been confirmed in East Asian waters, including those of South Korea and Japan, as well as in Chinese coastal waters [31,34,37]. Considering that jellyfish range expansion can generate various socio-economic impacts worldwide [4,28], further ecological studies on *C. pacifica* based on the baseline data presented in this study, together with predictions and assessments of the potential effects of future range expansion on marine ecosystems in East Asian waters, are needed.

These findings provide important baseline ecological information for understanding the early life history of *C. pacifica* and may contribute to predicting jellyfish population dynamics in East Asian waters under future environmental change. Furthermore, jellyfish blooms can substantially influence marine ecosystems by altering trophic interactions, increasing predation pressure on zooplankton, and competing with fish larvae for food resources, thereby potentially affecting fish recruitment and ecosystem stability [82,83]. In addition, such blooms can have significant socioeconomic impacts, including reduced fisheries productivity, damage to fishing gear, and negative effects on coastal industries and tourism [84,85]. Therefore, understanding the environmental drivers affecting early life stages, such as the ephyra stage, is essential for predicting and managing future jellyfish bloom events.

## 5. Conclusions

This study evaluated the effects of five temperature conditions (12, 16, 20, 24, and 28 °C) on the growth, feeding activity, and survival of *Chrysaora pacifica* ephyrae to identify their optimal thermal range. The results showed that 20–24 °C provided favorable conditions, supporting stable growth and feeding activity together with high survival (~90%), indicating optimal performance during this stage. In contrast, although growth and feeding activity increased at 28 °C, survival declined sharply to 22.5%, suggesting that elevated temperature may impose physiological stress. At 12 °C, survival was also relatively low (32.5%), accompanied by reduced growth and feeding activity, indicating metabolic limitation under low-temperature conditions. Overall, these findings identify temperature as a key driver of early life-stage performance in *C. pacifica* and suggest that suitable thermal conditions may play an important role in recruitment success and potential distribution in East Asian waters. This study provides essential baseline ecological information and highlights the need for further research integrating multiple environmental factors, such as salinity variability and interspecific food competition, to improve predictions of future population dynamics.

## Figures and Tables

**Table 1:** Mean size increase (mm) of *C. pacifica* ephyrae reared under five temperature conditions (12, 16, 20, 24, and 28 °C) over a 20-day period. Values are presented as mean ± standard deviation (SD), with 95% confidence intervals (CI). At each sampling point, 10 individuals were randomly selected from each of two replicate tanks per treatment (*n* = 20). Different letters (a–d) indicate significant differences among temperature treatments were assessed using Tukey’s post hoc test (*p* < 0.05).

Temperature (°C)	Salinity (PSU)	Growth (Size Increase, mm, *n* = 20)		Lower 95% CI	Upper 95% CI
		Mean	SD		
12	33	2.60 ^a^	0.17	2.1	3.0
16	33	3.05 ^b^	0.39	2.2	3.6
20	33	3.44 ^c^	0.59	2.3	4.5
24	33	3.42 ^c^	0.61	2.2	4.4
28	33	3.95 ^d^	0.81	2.3	5.3

**Table 2:** Mean daily growth rate (% d^−1^) of *C. pacifica* ephyrae reared under five temperature conditions (12, 16, 20, 24, and 28 °C) over a 20-day period. Values are presented as means with 95% confidence intervals (CI). At each sampling point, 10 individuals were randomly selected from each of two replicate tanks per treatment (*n* = 20). Different letters (a–d) indicate significant differences among temperature treatments were assessed using Tukey’s post hoc test (*p* < 0.05).

Temperature (°C)	Salinity (PSU)	Growth Rate (%, d^−1^), *n* = 20	Lower 95% CI	Upper 95% CI
12	33	3.24 ^a^	1.79	5.10
16	33	6.42 ^b^	5.31	7.78
20	33	8.84 ^c^	7.57	10.60
24	33	9.22 ^c^	7.57	10.94
28	33	12.1 ^d^	10.33	13.18

**Table 3:** Results of the generalized linear mixed models (GLMMs) analysis evaluating the effects of temperature, day, and their interaction on the size increase in *C. pacifica* ephyrae. The main effect of day and the temperature × day interaction were statistically significant. Pairwise comparisons for conditions showing significant differences were performed using Tukey’s post hoc test (*p* < 0.05).

Source	Growth (Size Increase, mm)			
	Estimate	S.E.	*t*-Value	*p*-Value
Temperature	0.0012	0.0024	0.0773	0.939
Days (Time)	−0.0456	0.0020	−23.1814	<0.001
Temperature × Days	0.0064	0.0011	65.5922	<0.001

**Table 4:** Mean feeding activity of *C. pacifica* ephyrae reared under five temperature conditions (12, 16, 20, 24, and 28 °C) over a 20-day experimental period, defined as the number of prey items observed in the gastric cavity per individual after 1 h of exposure. Values are presented as mean ± SD with 95% confidence intervals (CI). At each sampling point, 10 individuals were randomly selected from each of two replicate tanks per treatment (*n* = 20). This metric represents a short-term gut-content proxy rather than a true ingestion or clearance rate. Different superscript letters indicate significant differences among temperature treatments according to Tukey’s post hoc test (*p* < 0.05).

Temperature (°C)	Salinity (PSU)	Feeding Activity (prey ind^−1^ h^−1^)		Lower 95% CI	Upper 95% CI
		Mean	SD		
12	33	0.41 ^a^	0.521	0.0	2.0
16	33	2.47 ^b^	0.644	1.0	4.0
20	33	4.47 ^c^	0.521	3.0	6.0
24	33	4.59 ^c^	0.738	3.0	6.0
28	33	5.27 ^d^	0.521	5.0	7.0

**Table 5:** Results of the generalized linear mixed model (GLMM) analysis evaluating the effects of temperature, day, and their interaction on the feeding activity of *C. pacifica* ephyrae (prey ind^−1^ h^−1^). The main effect of temperature was statistically significant, whereas the effects of day and the temperature × day interaction were not. Pairwise comparisons among temperature treatments were conducted using Tukey’s post hoc test (*p* < 0.05).

Source	Feeding Activity (prey ind^−1^ h^−1^)			
	Estimate	S.E.	*t*-Value	*p*-Value
Temperature	0.3041	0.0134	22.7419	<0.001
Days (Time)	0.0001	0.0107	0.0070	0.934
Temperature × Days	−0.0010	0.0005	−1.8738	0.061

**Table 6:** Kaplan–Meier survival rates of *C. pacifica* ephyrae reared under five temperature conditions (12, 16, 20, 24, and 28 °C) over a 20-day period. Observed mortality events and 95% confidence intervals (CI) are included. Differences among temperature treatments were evaluated using the log-rank test (χ^2^ = 70.614, df = 4, *p* < 0.001). This analysis was based on pooled individual data for each treatment and did not explicitly account for potential clustering effects within tanks.

Temperature (°C)	Salinity (PSU)	Survival Rate (%)	Observed Event	Lower 95% (CI)	Upper Limit (CI)
12	33	32.5	27	0.208	0.508
16	33	65	14	0.518	0.816
20	33	90	4	0.812	0.998
24	33	87.5	5	0.778	0.984
28	33	22.5	31	0.127	0.400

## Data Availability

The data presented in this study are available from the corresponding author upon reasonable request. The data are included within the article and its Supplementary Materials.

## Supplementary Materials

The following supporting information can be downloaded at: https://www.mdpi.com/article/10.3390/biology15080597/s1, Table S1: Results of Tukey’s post hoc comparisons assessing the temperature × day interaction effects on the size increase of *C. pacifica* ephyrae under five different temperature conditions (12, 16, 20, 24, and 28 °C). Comparisons were conducted at 2-day intervals over a 20-day experimental period. Statistically significant differences were found in most pairwise comparisons (*p* < 0.001), except between 20 °C and 24 °C from Day 4 onward (*p* > 0.05); Table S2: Tukey’s post hoc comparisons assessing the main effect of temperature on the feeding activity of *C. pacifica* ephyrae reared under five different temperature conditions (12, 16, 20, 24, and 28 °C). Statistically significant differences were found among all temperature pairs (*p* < 0.001), except between 20 °C and 24 °C (*p* = 0.801), where no significant difference was observed. The highest feeding activity was recorded at 28 °C; Video S1: Time-lapse video showing the strobilation process of *Chrysaora pacifica* polyps.

## References

[1] Levitus S., Antonov J.I., Boyer T.P., Locarnini R.A., Garcia H., Mishonov A.V. (2009). Global ocean heat content 1955–2008 in light of recently revealed instrumentation problems. Geophys. Res. Lett..

[2] Doney S.C., Ruckelshaus M., Duffy J.E., Barry J.P., Chan F., English C.A., Galindo H.M., Grebmeier J.M., Hollowed A.B., Knowlton N. (2012). Climate change impacts on marine ecosystems. Annu. Rev. Mar. Sci..

[3] Treible L.M., Condon R.H. (2019). Temperature-driven asexual reproduction and strobilation in three scyphozoan jellyfish polyps. J. Exp. Mar. Biol. Ecol..

[4] Lee S.-H., Scotti M., Jung S., Hwang J.-S., Molinero J.C. (2023). Jellyfish blooms challenge the provisioning of ecosystem services in Korean coastal waters. Hydrobiologia.

[5] Venegas R.M., Acevedo J., Treml E.A. (2023). Three decades of ocean warming impacts on marine ecosystems: A review and perspective. Deep Sea Res. Part II Top. Stud. Oceanogr..

[6] Wang F., Meng Q., Tang X., Hu D. (2013). The long-term variability of sea surface temperature in the seas east of China in the past 40 years. Acta Oceanol. Sin..

[7] Han I.-S., Lee J.-S. (2020). Change in the annual amplitude of sea surface temperature due to climate change in the recent decade around the Korean Peninsula. J. Korean Soc. Mar. Environ. Saf..

[8] Poloczanska E.S., Brown C.J., Sydeman W.J., Kiessling W., Schoeman D.S., Moore P.J., Brander K., Bruno J.F., Buckley L.B., Burrows M.T. (2013). Global imprint of climate change on marine life. Nat. Clim. Change.

[9] Richardson A.J., Bakun A., Hays G.C., Gibbons M.J. (2009). The jellyfish joyride: Causes, consequences and management responses to a more gelatinous future. Trends Ecol. Evol..

[10] Purcell J.E., Uye S., Lo W.-T. (2007). Anthropogenic causes of jellyfish blooms and their direct consequences for humans: A review. Mar. Ecol. Prog. Ser..

[11] Purcell J.E., Atienza D., Fuentes V., Olariaga A., Tilves U., Colahan C., Gili J.-M. (2012). Temperature effects on asexual reproduction rates of scyphozoan species from the northwest Mediterranean Sea. Hydrobiologia.

[12] Xie C., Fan M., Wang X., Chen M. (2015). Dynamic model for life history of Scyphozoa. PLoS ONE.

[13] Xing Y., Liu Q., Zhang M., Zhen Y., Mi T., Yu Z. (2020). Effects of temperature and salinity on the asexual reproduction of *Aurelia coerulea* polyps. J. Oceanol. Limnol..

[14] Schäfer S., Gueroun S.K.M., Andrade C.A.P., Canning-Clode J. (2021). Combined effects of temperature and salinity on polyps and ephyrae of *Aurelia solida* (Cnidaria: Scyphozoa). Diversity.

[15] Lee S.-H., Hwang J.-S., Kim K.-Y., Molinero J.C. (2021). Contrasting effects of regional and local climate on the interannual variability and phenology of the scyphozoan *Aurelia coerulea* and *Nemopilema nomurai* in the Korean Peninsula. Diversity.

[16] Gershwin L.-A. (2013). Stung!: On Jellyfish Blooms and the Future of the Ocean.

[17] Fu Z., Li J., Wang J., Lai J., Liu Y., Sun M. (2020). Combined effects of temperature and salinity on the growth and pulsation of moon jellyfish (*Aurelia coerulea*) ephyrae. Am. J. Life Sci..

[18] Condon R.H., Duarte C.M., Pitt K.A., Robinson K.L., Lucas C.H., Sutherland K.R., Mianzan H.W., Bogeberg M., Purcell J.E., Decker M.B. (2013). Recurrent jellyfish blooms are a consequence of global oscillations. Proc. Natl. Acad. Sci. USA.

[19] Sanz-Martín M., Pitt K.A., Condon R.H., Lucas C.H., Novaes de Santana C., Duarte C.M. (2016). Flawed citation practices facilitate the unsubstantiated perception of a global trend toward increased jellyfish blooms. Glob. Ecol. Biogeogr..

[20] Quiñones J., Mianzan H., Purca S., Robinson K.L., Adams G.D., Acha M.A. (2015). Climate-driven population size fluctuations of jellyfish (*Chrysaora plocamia*) off Peru. Mar. Biol..

[21] Graham W.M., Gelcich S., Robinson K.L., Duarte C.M., Brotz L., Purcell J.E., Madin L.P., Mianzan H.W., Sutherland K.R., Uye S.-i. (2014). Linking human well-being and jellyfish: Ecosystem services, impacts, and societal responses. Front. Ecol. Environ..

[22] Yue M., Mi T., Li Y., Gong X., Zhen Y. (2023). Molecular monitoring plankton community during jellyfish blooms in the water near the Liaoning Hongyan River Nuclear Power Plant. J. Oceanol. Limnol..

[23] Khong N.M.H., Yusoff F.M., Bakar J., Basri M., Ismail M., Chan K.W., Nishikawa J. (2016). Nutritional composition and total collagen content of three commercially important edible jellyfish. Food Chem..

[24] Fernández Alías A., Montaño Barroso T., Conde Caño M.R., Manchado Pérez S., López Galindo C., Quispe Becerra J.I., Marcos C., Pérez Ruzafa A. (2022). Nutrient overload promotes the transition from top down to bottom-up control and triggers dystrophic crises in a Mediterranean coastal lagoon. Sci. Total Environ..

[25] Pulcinella J., Bonanomi S., Colombelli A., Fortuna C.M., Moro F., Lucchetti A., Sala A. (2019). Bycatch of Loggerhead Turtle (*Caretta caretta*) in the Italian Adriatic Midwater Pair Trawl Fishery. Front. Mar. Sci..

[26] Fernández Alías A., Marcos C., Pérez Ruzafa A. (2021). Larger scyphozoan species dwelling in temperate, shallow waters show higher blooming potential. Mar. Pollut. Bull..

[27] Sun T., Luo Z., Peng S., Schiariti A., Du C., Zhao J., Dong Z. (2023). Physiological response of *Aurelia coerulea* polyps to elevated seasonal temperatures. Hydrobiologia.

[28] Fernández Alías A., Marcos C., Pérez Ruzafa A. (2024). The unpredictability of scyphozoan jellyfish blooms. Front. Mar. Sci..

[29] Scorrano S., Aglieri G., Boero F., Dawson M.N., Piraino S. (2017). Unmasking *Aurelia* species in the Mediterranean Sea: An integrative morphometric and molecular approach. Zool. J. Linn. Soc..

[30] Feng S., Wang S.-W., Sun S., Zhang F., Zhang G.-T., Liu M.-T., Uye S.-i. (2018). Strobilation of three scyphozoans (*Aurelia coerulea*, *Nemopilema nomurai* and *Rhopilema esculentum*) in the field at Jiaozhou Bay, China. Mar. Ecol. Prog. Ser..

[31] Seo Y., Kim D.-H., Chae J., Ki J.-S. (2020). Distribution of the sea nettle *Chrysaora pacifica* (Goette, 1886) (Semaeostomeae; Pelagiidae) in Korea using molecular markers. Ocean Polar Res..

[32] Kitajima S., Hasegawa T., Nishiuchi K., Kiyomoto Y., Taneda T., Yamada H. (2020). Temporal fluctuations in abundance and size of the giant jellyfish *Nemopilema nomurai* medusae in the northern East China Sea, 2006–2017. Mar. Biol..

[33] Péron F., Lesueur C.A. (1810). Tableau des caractères génériques et spécifiques de toutes les espèces de méduses connues jusqu’à ce jour. Ann. Mus. Natl. Hist. Nat. Paris.

[34] Lee H.E., Yoon W., Chae J., Ki J.-S. (2016). Re-description of *Chrysaora pacifica* (Goette, 1886) (Cnidaria, Scyphozoa) from Korean coastal waters: Morphology and molecular comparisons. Ocean Polar Res..

[35] Chae J., Seo Y., Yu W.B., Yoon W.D., Lee H.E., Chang S.-J., Ki J.-S. (2018). Comprehensive analysis of the jellyfish *Chrysaora pacifica* (Goette, 1886) (Semaeostomeae: Pelagiidae) with description of the complete rDNA sequence. Zool. Stud..

[36] Takasu H., Inomata H., Uchino K., Tahara S., Mori K., Hirano Y., Harada K., Yamaguchi M., Nozoe Y., Akiyama H. (2019). Spatio-temporal distribution of environmental DNA derived from Japanese sea nettle jellyfish *Chrysaora pacifica* in Omura Bay, Kyushu, Japan. Plankton Benthos Res..

[37] Wang Y., Wang N., Sun S., Wang J., Jin X. (2022). First record of the non-native jellyfish *Chrysaora pacifica* (Goette, 1886) (Cnidaria, Scyphozoa) in Liaodong Bay, Bohai Sea, China. BioInvasions Rec..

[38] Morandini A.C., Marques A.C. (2010). Revision of the genus *Chrysaora* Péron & Lesueur, 1810 (Cnidaria: Scyphozoa). Zootaxa.

[39] Toyokawa M. (2011). First record of wild polyps of *Chrysaora pacifica* (Goette, 1886) (Scyphozoa, Cnidaria). Plankton Benthos Res..

[40] Park J.-H. (2002). Two new records of Siphonophora (Hydrozoa) and Semaeostomeae (Scyphozoa) in Korea. Anim. Syst. Evol. Divers..

[41] Uchida T. (1970). Medusan collection deposited in the Iwa Marine Biological Station, the Manazuru Peninsula. Publ. Seto Mar. Biol. Lab..

[42] Kinoshita J., Hiromi J., Yamada Y. (2006). Abundance and biomass of scyphomedusae, *Aurelia aurita* and *Chrysaora melanaster*, and Ctenophora, *Bolinopsis mikado*, with estimates of their feeding impact on zooplankton in Tokyo Bay, Japan. J. Oceanogr..

[43] Ikawa Y., Fujita N., Yachi Y., Inoue N., Kato A., Kuroda M., Yachie A. (2016). Life-threatening complications of jellyfish *Chrysaora pacifica* stings in a 5-year-old child. Br. J. Dermatol..

[44] Shin K.-H., Choi K.-H. (2025). Effects of the seawater temperature and salinity on the survival and growth of the *Sanderia malayensis* (Cnidaria: Scyphozoa) ephyrae. Mar. Biol. Res..

[45] Thein H., Ikeda H., Uye S. (2013). Ecophysiological characteristics of podocysts in *Chrysaora pacifica* (Goette) and *Cyanea nozakii* Kishinouye (Cnidaria: Scyphozoa: Semaeostomeae): Effects of environmental factors on their production, dormancy and excystment. J. Exp. Mar. Biol. Ecol..

[46] Takao M., Uye S.-i. (2018). Effects of low salinity on the physiological ecology of planulae and polyps of scyphozoans in the East Asian Marginal Seas: Potential impacts of monsoon rainfall on medusa population size. Hydrobiologia.

[47] Båmstedt U., Lane J., Martinussen M.B. (1999). Bioenergetics of ephyra larvae of the scyphozoan jellyfish *Aurelia aurita* in relation to temperature and salinity. Mar. Biol..

[48] Widmer C.L. (2015). Influences of Temperature and Salinity on Asexual Reproduction and Development of Scyphozoan Jellyfish from the British Isles. Ph.D. Thesis.

[49] Kamiyama T. (2018). Planktonic ciliates as food for the scyphozoan *Aurelia coerulea*: Feeding and growth responses of ephyra and metephyra stages. J. Oceanogr..

[50] Gambill M., Peck M.A. (2014). Respiration rates of the polyps of four jellyfish species: Potential thermal triggers and limits. J. Exp. Mar. Biol. Ecol..

[51] Shin K.-H., Choi K.-H. (2025). The effects of salinity on the growth, survival, and feeding of *Sanderia malayensis* (Cnidaria: Scyphozoa) ephyrae. Diversity.

[52] Riisgård H.U. (2022). Superfluous feeding and growth of jellyfish *Aurelia aurita*. J. Mar. Sci. Eng..

[53] Straehler-Pohl I., Jarms G. (2010). Identification key for young ephyrae: A first step for early detection of jellyfish blooms. Hydrobiologia.

[54] Bolker B.M., Brooks M.E., Clark C.J., Geange S.W., Poulsen J.R., Stevens M.H.H., White J.-S.S. (2009). Generalized linear mixed models: A practical guide for ecology and evolution. Trends Ecol. Evol..

[55] Bates D., Mächler M., Bolker B., Walker S. (2015). Fitting linear mixed-effects models using lme4. J. Stat. Softw..

[56] Kaplan E.L., Meier P. (1958). Nonparametric estimation from incomplete observations. J. Am. Stat. Assoc..

[57] Therneau T.M., Grambsch P.M. (2000). Modeling Survival Data: Extending the Cox Model.

[58] Kassambara A., Kosinski M., Biecek P., Scheipl S. (2021). survminer: Drawing Survival Curves Using ‘ggplot2’ (R Package Version 0.4.9). CRAN. https://CRAN.R-project.org/package=survminer.

[59] Uye S.-i., Ueta U. (2004). Recent increases of jellyfish populations and their nuisance to fisheries in the Inland Sea of Japan. Bull. Jpn. Soc. Fish. Oceanogr..

[60] Yoshioka N., Kamizono M. (2005). Recent increase of jellyfish populations and their nuisance to fisheries in the Buzen-Sea. Bull. Fukuoka Fish. Mar. Technol. Res. Cent..

[61] Yamashita O., Sakiyama T. (1999). Medusae collected in Enoshima-Shonan port and its adjacent waters. Nat. Hist. Rep. Kanagawa.

[62] Sakiyama T., Adachi A. (2001). Medusae collected in Enoshima-Shonan port and its adjacent waters—II. Nat. Hist. Rep. Kanagawa.

[63] Fu Z., Shibata M., Makabe R., Ikeda H., Uye S.-i. (2014). Body size reduction under starvation, and the point of no return, in ephyrae of the moon jellyfish *Aurelia aurita*. Mar. Ecol. Prog. Ser..

[64] Purcell J.E., White J.R., Nemazie D.A., Wright D.A. (1999). Temperature, salinity and food effects on asexual reproduction and abundance of the scyphozoan *Chrysaora quinquecirrha*. Mar. Ecol. Prog. Ser..

[65] Widmer C.L. (2005). Effects of temperature on growth of north-east Pacific moon jellyfish ephyrae, *Aurelia labiata* (Cnidaria: Scyphozoa). J. Mar. Biol. Assoc. UK.

[66] Kawahara M., Uye S.-i., Ohtsu K., Iizumi H. (2006). Unusual population explosion of the giant jellyfish *Nemopilema nomurai* (Scyphozoa: Rhizostomeae) in East Asian waters. Mar. Ecol. Prog. Ser..

[67] Han C.-H., Uye S.-i. (2010). Combined effects of food supply and temperature on asexual reproduction and somatic growth of polyps of the common jellyfish *Aurelia aurita* s.l. Plankton Benthos Res..

[68] Sun M., Dong J., Purcell J.E., Li Y., Duan Y., Wang A., Wang B. (2015). Testing the influence of previous-year temperature and food supply on development of *Nemopilema nomurai* blooms. Hydrobiologia.

[69] Makabe R., Takeoka H., Uye S.-i. (2015). Offshore dispersion of ephyrae and medusae of *Aurelia aurita* s.l. (Cnidaria: Scyphozoa) from port enclosures: Physical and biological factors. J. Mar. Syst..

[70] Avian M., Motta G., Prodan M., Tordoni E., Macaluso V., Beran A., Goruppi A., Bacaro G., Tirelli V. (2021). Asexual reproduction and strobilation of *Sanderia malayensis* (Scyphozoa, Pelagiidae) in relation to temperature: Experimental evidence and implications. Diversity.

[71] Uchida T., Sugiura Y. (1975). On the ephyra and postephyra of Semaeostome medusa, *Sanderia malayensis* Goette, 1886. J. Fac. Sci. Hokkaido Univ. Ser. VI Zool..

[72] Minemizu R., Kubota S., Hirano Y., Lindsay D.J. (2015). A Photographic Guide to the Jellyfishes of Japan.

[73] Toshino S., Yamashiro H., Tanimoto M. (2018). New record of *Sanderia malayensis* from the Ryukyu Archipelago, Southern Japan. Fauna Ryukyuana.

[74] Gassen L., Esters L., Ribas Ribas M., Wurl O. (2024). The impact of rainfall on the sea surface salinity: A mesocosm study. Sci. Rep..

[75] Arcodia M., Barnes E.A., Durack P.J., Keys P.W., Rocha J. (2025). Sea surface salinity provides subseasonal predictability for forecasts of opportunity of U.S. summertime precipitation. J. Geophys. Res. Atmos..

[76] Yu S., Song D., Fan M., Xie C. (2023). Effects of temperature and salinity on growth of *Aurelia aurita*. Ecol. Model..

[77] Schiariti A., Morandini A.C., Jarms G., Paes R.V.G., Franke S., Mianzan H. (2014). Asexual reproduction strategies and blooming potential in Scyphozoa. Mar. Ecol. Prog. Ser..

[78] Fernández-Alías A., Molinero J.C., Ismael J., Bonnet D., Marcos C., Perez-Ruzafa A. (2023). Phenology of scyphozoan jellyfish species in a eutrophication and climate change context. Mar. Pollut. Bull..

[79] Purcell J.E., Baxter E.J., Fuentes V.L., Allan G., Burnell G. (2013). Jellyfish as products and problems of aquaculture. Advances in Aquaculture Hatchery Technology.

[80] Frolova A., Miglietta M.P. (2020). Insights on bloom forming jellyfish (Class: Scyphozoa) in the Gulf of Mexico: Environmental tolerance ranges and limits suggest differences in habitat preference and resistance to climate change among congeners. Front. Mar. Sci..

[81] Feng S., Sun S., Li C., Zhang F. (2022). Controls of *Aurelia coerulea* and *Nemopilema nomurai* (Cnidaria: Scyphozoa) blooms in the coastal sea of China: Strategies and measures. Front. Mar. Sci..

[82] Wang P., Zhang F., Guo D., Chi X., Feng S., Sun S. (2024). Trophic effects of jellyfish blooms on fish populations in ecosystems of the coastal waters of China. Sci. Total Environ..

[83] Stoltenberg I., Dierking J., Müller-Navarra D.C., Javidpour J. (2021). Review of jellyfish trophic interactions in the Baltic Sea. Mar. Biol. Res..

[84] Bosch-Belmar M., Milisenda G., Basso L., Doyle T.K., Leone A., Piraino S. (2020). Jellyfish impacts on marine aquaculture and fisheries. Rev. Fish. Sci. Aquac..

[85] Ruiz-Frau A. (2022). Impacts of jellyfish presence on tourists’ holiday destination choices and their willingness to pay for mitigation measures. J. Environ. Plan. Manag..

