# Supplementary material for: The Effects of Temperature on the Growth, Survival, and Feeding of Chrysaora pacifica (Cnidaria: Scyphozoa) Ephyrae
Source: Biology (Basel). 2026 Apr 9;15(8):597. doi: 10.3390/biology15080597 (PMC13113277; doi:10.3390/biology15080597)
Supplement: Supplementary file 1 [file biology-15-00597-s001.zip › Supplementary Materials (Table S1, Table S2).pdf]

Table S1. Results of Tukey's post hoc comparisons assessing the temperature × day interaction effects on the size increase of *C. pacifica* ephyrae under five different temperature conditions (12, 16, 20, 24, and 28 °C). Comparisons were conducted at 2-day intervals over a 20-day experimental period. Statistically significant differences were found in most pairwise comparisons ($p < 0.001$), except between 20 °C and 24 °C from Day 4 onward ($p > 0.05$).

Comparison		Estimate	S.E.	df	*t-Value*	*p-Value*
Days (Time)	Temperature (°C)					
2	12 - 16	-0.075	0.026	1071.239	-2.884	0.033
2	12 - 20	-0.270	0.026	1071.239	-10.382	<.001
2	12 - 24	-0.195	0.026	1071.239	-7.498	<.001
2	12 - 28	-0.185	0.026	1071.250	-7.114	<.001
2	16 - 20	-0.195	0.026	1071.239	-7.498	<.001
2	16 - 24	-0.120	0.026	1071.239	-4.614	<.001
2	16 - 28	-0.110	0.026	1071.250	-4.230	<.001
2	20 - 24	0.075	0.026	1071.239	2.884	0.033
2	20 - 28	0.085	0.026	1071.250	3.269	0.010
2	24 - 28	0.010	0.026	1071.250	0.385	0.995
4	12 - 16	-0.250	0.026	1071.239	-9.613	<.001
4	12 - 20	-0.435	0.026	1071.239	-16.727	<.001
4	12 - 24	-0.450	0.026	1071.239	-17.304	<.001
4	12 - 28	-0.425	0.026	1071.250	-16.343	<.001
4	16 - 20	-0.185	0.026	1071.239	-7.114	<.001
4	16 - 24	-0.200	0.026	1071.239	-7.691	<.001
4	16 - 28	-0.175	0.026	1071.250	-6.729	<.001
4	20 - 24	-0.015	0.026	1071.239	-0.577	0.978
4	20 - 28	0.010	0.026	1071.250	0.385	0.995
4	24 - 28	0.025	0.026	1071.250	0.961	0.872

6	12 - 16	-0.250	0.026	1071.239	-9.613	<.001
6	12 - 20	-0.480	0.026	1071.239	-18.458	<.001
6	12 - 24	-0.530	0.026	1071.239	-20.380	<.001
6	12 - 28	-0.555	0.026	1071.250	-21.342	<.001
6	16 - 20	-0.230	0.026	1071.239	-8.844	<.001
6	16 - 24	-0.280	0.026	1071.239	-10.767	<.001
6	16 - 28	-0.305	0.026	1071.250	-11.728	<.001
6	20 - 24	-0.050	0.026	1071.239	-1.923	0.306
6	20 - 28	-0.075	0.026	1071.250	-2.884	0.033
6	24 - 28	-0.025	0.026	1071.250	-0.961	0.872
8	12 - 16	-0.435	0.026	1071.239	-16.727	<.001
8	12 - 20	-0.685	0.026	1071.239	-26.341	<.001
8	12 - 24	-0.675	0.026	1071.239	-25.956	<.001
8	12 - 28	-0.835	0.026	1071.250	-32.109	<.001
8	16 - 20	-0.250	0.026	1071.239	-9.613	<.001
8	16 - 24	-0.240	0.026	1071.239	-9.229	<.001
8	16 - 28	-0.400	0.026	1071.250	-15.381	<.001
8	20 - 24	0.010	0.026	1071.239	0.385	0.995
8	20 - 28	-0.150	0.026	1071.250	-5.768	<.001
8	24 - 28	-0.160	0.026	1071.250	-6.153	<.001
10	12 - 16	-0.535	0.026	1071.239	-20.573	<.001
10	12 - 20	-0.860	0.026	1071.239	-33.070	<.001
10	12 - 24	-0.890	0.026	1071.239	-34.223	<.001
10	12 - 28	-1.115	0.026	1071.250	-42.875	<.001

10	16 - 20	-0.325	0.026	1071.239	-12.497	<.001
10	16 - 24	-0.355	0.026	1071.239	-13.651	<.001
10	16 - 28	-0.580	0.026	1071.250	-22.303	<.001
10	20 - 24	-0.030	0.026	1071.239	-1.154	0.778
10	20 - 28	-0.255	0.026	1071.250	-9.806	<.001
10	24 - 28	-0.225	0.026	1071.250	-8.652	<.001
12	12 - 16	-0.605	0.026	1071.239	-23.264	<.001
12	12 - 20	-1.050	0.026	1071.239	-40.376	<.001
12	12 - 24	-1.030	0.026	1071.239	-39.607	<.001
12	12 - 28	-1.350	0.026	1071.250	-51.912	<.001
12	16 - 20	-0.445	0.026	1071.239	-17.112	<.001
12	16 - 24	-0.425	0.026	1071.239	-16.343	<.001
12	16 - 28	-0.745	0.026	1071.250	-28.648	<.001
12	20 - 24	0.020	0.026	1071.239	0.769	0.939
12	20 - 28	-0.300	0.026	1071.250	-11.536	<.001
12	24 - 28	-0.320	0.026	1071.250	-12.305	<.001
14	12 - 16	-0.655	0.026	1071.239	-25.187	<.001
14	12 - 20	-1.210	0.026	1071.239	-46.529	<.001
14	12 - 24	-1.165	0.026	1071.239	-44.798	<.001
14	12 - 28	-1.545	0.026	1071.250	-59.410	<.001
14	16 - 20	-0.555	0.026	1071.239	-21.342	<.001
14	16 - 24	-0.510	0.026	1071.239	-19.611	<.001
14	16 - 28	-0.890	0.026	1071.250	-34.223	<.001
14	20 - 24	0.045	0.026	1071.239	1.730	0.416

14	20 - 28	-0.335	0.026	1071.250	-12.882	<.001
14	24 - 28	-0.380	0.026	1071.250	-14.612	<.001
16	12 - 16	-0.660	0.026	1071.239	-25.379	<.001
16	12 - 20	-1.210	0.026	1071.239	-46.529	<.001
16	12 - 24	-1.190	0.026	1071.239	-45.759	<.001
16	12 - 28	-1.780	0.029	1097.180	-62.258	<.001
16	16 - 20	-0.550	0.026	1071.239	-21.149	<.001
16	16 - 24	-0.530	0.026	1071.239	-20.380	<.001
16	16 - 28	-1.120	0.029	1097.180	-39.170	<.001
16	20 - 24	0.020	0.026	1071.239	0.769	0.939
16	20 - 28	-0.570	0.029	1097.180	-19.930	<.001
16	24 - 28	-0.590	0.029	1097.180	-20.629	<.001
18	12 - 16	-0.675	0.026	1071.239	-25.956	<.001
18	12 - 20	-1.315	0.026	1071.239	-50.566	<.001
18	12 - 24	-1.315	0.026	1071.239	-50.566	<.001
18	12 - 28	-2.033	0.031	1111.255	-66.137	<.001
18	16 - 20	-0.640	0.026	1071.239	-24.610	<.001
18	16 - 24	-0.640	0.026	1071.239	-24.610	<.001
18	16 - 28	-1.358	0.031	1111.255	-44.179	<.001
18	20 - 24	<.001	0.026	1071.239	<.001	1.000
18	20 - 28	-0.718	0.031	1111.255	-23.360	<.001
18	24 - 28	-0.718	0.031	1111.255	-23.360	<.001
20	12 - 16	-0.705	0.026	1071.239	-27.110	<.001
20	12 - 20	-1.485	0.026	1071.239	-57.103	<.001

20	12 - 24	-1.420	0.026	1071.239	-54.604	<.001
20	12 - 28	-2.271	0.033	1120.339	-69.205	<.001
20	16 - 20	-0.780	0.026	1071.239	-29.994	<.001
20	16 - 24	-0.715	0.026	1071.239	-27.494	<.001
20	16 - 28	-1.566	0.033	1120.339	-47.724	<.001
20	20 - 24	0.065	0.026	1071.239	2.499	0.091
20	20 - 28	-0.786	0.033	1120.339	-23.959	<.001
20	24 - 28	-0.851	0.033	1120.339	-25.940	<.001

Note. p-values were adjusted using Tukey's test to correct for multiple comparison

Table S2. Tukey's post hoc comparisons assessing the main effect of temperature on the feeding activity of *C. pacifica* ephyrae reared under five different temperature conditions (12, 16, 20, 24, and 28 °C). Statistically significant differences were found among all temperature pairs ($p < 0.001$), except between 20 °C and 24 °C ($p = 0.801$), where no significant difference was observed. The highest feeding activity was recorded at 28 °C.

Comparison	Estimate	S.E.	df	*t*-Value	*p*-Value
Temperature (°C)					
12 - 16	-2.055	0.106	103.789	-19.308	<.001
12 - 20	-4.055	0.106	103.789	-38.104	<.001
12 - 24	-4.173	0.106	103.789	-39.215	<.001
12 - 28	-4.857	0.108	110.262	-44.910	<.001
16 - 20	-2.000	0.106	103.789	-18.796	<.001
16 - 24	-2.118	0.106	103.789	-19.906	<.001
16 - 28	-2.803	0.108	110.262	-25.914	<.001
20 - 24	-0.118	0.106	103.789	-1.111	0.801
20 - 28	-0.803	0.108	110.262	-7.423	<.001
24 - 28	-0.685	0.108	110.262	-6.330	<.001

Note. p-values were adjusted using Tukey's test to correct for multiple comparison.
